# Representing true plant genomes: haplotype-resolved hybrid pepper genome with trio-binning

**DOI:** 10.3389/fpls.2023.1184112

**Published:** 2023-11-16

**Authors:** Emily E. Delorean, Ramey C. Youngblood, Sheron A. Simpson, Ashley N. Schoonmaker, Brian E. Scheffler, William B. Rutter, Amanda M. Hulse-Kemp

**Affiliations:** ^1^ Genomics and Bioinformatics Research Unit, USDA-ARS, Raleigh, NC, United States; ^2^ Crop and Soil Sciences Department, North Carolina State University, Raleigh, NC, United States; ^3^ Institute for Genomics, Biocomputing and Biotechnology, Mississippi State University, Starkville, MS, United States; ^4^ Genomics and Bioinformatics Research Unit, United States Department of Agriculture - Agriculture Research Service (USDA-ARS), Stoneville, MS, United States; ^5^ US Vegetable Laboratory, United States Department of Agriculture - Agriculture Research Service (USDA-ARS), Charleston, SC, United States

**Keywords:** haplotype, pepper, genome assembly, trio-binning, HiFi

## Abstract

As sequencing costs decrease and availability of high fidelity long-read sequencing increases, generating experiment specific *de novo* genome assemblies becomes feasible. In many crop species, obtaining the genome of a hybrid or heterozygous individual is necessary for systems that do not tolerate inbreeding or for investigating important biological questions, such as hybrid vigor. However, most genome assembly methods that have been used in plants result in a merged single sequence representation that is not a true biologically accurate representation of either haplotype within a diploid individual. The resulting genome assembly is often fragmented and exhibits a mosaic of the two haplotypes, referred to as haplotype-switching. Important haplotype level information, such as causal mutations and structural variation is therefore lost causing difficulties in interpreting downstream analyses. To overcome this challenge, we have applied a method developed for animal genome assembly called trio-binning to an intra-specific hybrid of chili pepper (*Capsicum annuum* L. cv. HDA149 *x Capsicum annuum* L. cv. HDA330). We tested all currently available softwares for performing trio-binning, combined with multiple scaffolding technologies including Bionano to determine the optimal method of producing the best haplotype-resolved assembly. Ultimately, we produced highly contiguous biologically true haplotype-resolved genome assemblies for each parent, with scaffold N50s of 266.0 Mb and 281.3 Mb, with 99.6% and 99.8% positioned into chromosomes respectively. The assemblies captured 3.10 Gb and 3.12 Gb of the estimated 3.5 Gb chili pepper genome size. These assemblies represent the complete genome structure of the intraspecific hybrid, as well as the two parental genomes, and show measurable improvements over the currently available reference genomes. Our manuscript provides a valuable guide on how to apply trio-binning to other plant genomes.

## 1 Introduction

Reference genomes are now available for hundreds of plant species, providing valuable tools for researchers and plant breeders. However, there are still limitations in many of the available reference genomes. As the numbers of *de novo* reference assemblies increase and pan-genome assemblies become more widely available (Bayer et al., 2020), we are finding that individuals exhibit varying amounts of presence/absence variation (PAV), copy number variation (CNV) and structural variation (SV) (Liu et al., 2020; Wang et al., 2020; Lee et al., 2022; Tang et al., 2022; Yang et al., 2022; Zhou et al., 2022). This variation not only occurs between individuals, but also within the genome of a single individual when that individual is heterozygous. Important genetic information is often lost when sequencing reads are aligned to a single merged reference genome. Ideally, the true haplotype of each individual within a project would be available, particularly for founder parents of breeding lines.

The decreasing cost of DNA sequencing in conjunction with third generation long-read sequencing technologies has brought custom plant genome assemblies a step closer to reality, even for polyploids and species with large genomes (Kress et al., 2022; Newman et al., 2023; Sahu and Liu, 2023). However, there are still many technical hurdles involved in assembling a biologically accurate fully-phased plant genome. The typical genome assembly is a haploid representation of a diploid individual. If the individual is homozygous then a single haploid genome assembly is sufficient given that the two haploid genomes, or haplotypes, within the organism are effectively the same. If the organism is heterozygous then there becomes the chance that the resulting genome assembly is a mosaic or chimera of the individual’s two haplotypes (haplotype switching). These chimeric genomic regions are not biologically accurate and may be misleading in downstream analysis, such as during candidate gene mining (Benevenuto et al., 2019). Correctly assembling each haploid genome is referred to as haplotype phasing and is one of the key challenges facing modern genome assembly methods.

Advances in computational approaches can also help overcome these genome assembly challenges, significantly decrease costs, and improve the quality of the final assemblies. The error profiles in long-read sequencing were drastically improved with the availability of circular consensus or high-fidelity (HiFi) reads, which became available in 2019 (Wenger et al., 2019). Currently there are two genome assembly softwares that support HiFi reads, Hifiasm (Cheng et al., 2021) and HiCanu (Nurk et al., 2020). Hifiasm is an intrinsically haplotype-aware assembler that builds a string graph of overlapping sequences where all haplotype information is saved as a fork (called bubbles). Hifiasm by default also generates two partially phased haplotypes assemblies (hap1/hap2).

Several approaches have been used to try and correct haplotype switching and produce an accurate fully phased genome. Prior to HiFi reads, haplotype phasing was often highly involved and relied on single nucleotide polymorphism (SNP) data or germ cell sequencing (Minio et al., 2017; Shi et al., 2019; Campoy et al., 2020; Minio et al., 2022). Falcon and Falcon-Unzip assemblers corrected the high error rate of PacBio Continuous Long Read Sequencing (CLR) and used differences in SNPs to partition haplotypes (Chin et al., 2016). Another advance in technology came with the advent of Hi-C sequencing for scaffolding, which relies on intra-chromosomal contacts. The Hi-C paired-end reads are aligned to a partially phased genome assembly to determine which pieces of the assembly, or haplotigs, belong together along a chromosome. The disadvantage of using Hi-C is the absence of inter-chromosomal information, which means that sorting chromosomes into the proper genome isn’t possible and the phasing success may be lower compared to other methods (Kronenberg et al., 2021; Mao et al., 2023). While Bionano optical maps have the capability of providing haplotype phasing for humans (Seo et al., 2016), that utility is not yet available for plants.

**Figure 1:**
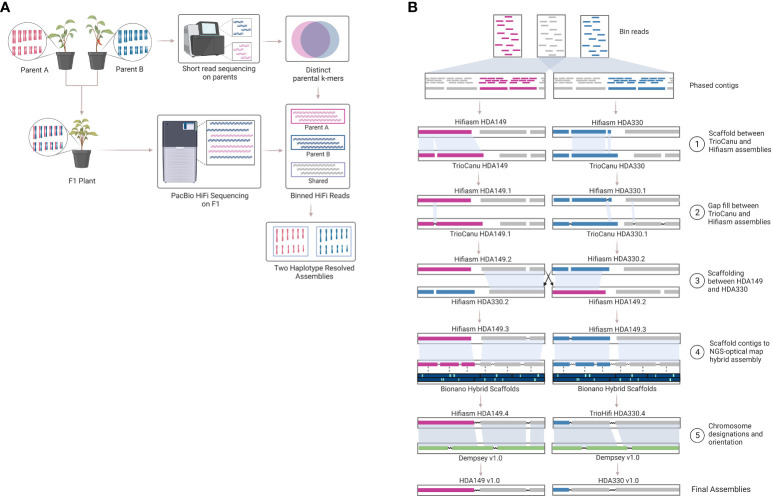
Trio-binning workflow. Overview of the trio-binning workflow used for producing haplotype-resolved biologically accurate plant genomes. **(A)** Schematic of lab-based and theoretical protocol utilized in the trio-binning workflow. **(B)** Detailed overview of the *in silico* process for assembly and scaffolding, including incorporation of Bionano optical mapping.

Another method to resolve haplotype switching is trio-binning, where short reads generated from the parents are used to bin long reads generated from the offspring prior to assembly. A ‘trio’ refers to the combination of a mother-father-offspring. This method has been suggested for development of telomere to telomere (T2T) or gapless genome assembly efforts (Nurk et al., 2022). Excitingly, we are seeing the first T2T plant genomes being released, but these again are for small homozygous genome species like rice and did not use trio-binning (Li et al., 2021; Gladman et al., 2023). The trio-binning genome assembly method (**Figure 1**) relies on HiFi long read sequencing of an F_1_ individual and short read sequencing of the two parent lines of the F_1_ individual (**Figure 1A**). The short reads from the parents are broken into k-mers that are distinct in one parent line compared to the other parental line. These k-mers are then aligned to the long reads to partition the long reads into 3 sequence bins containing either 1) long reads unique to parent A, 2) long reads unique to parent B, or 3) long reads that are likely shared between parent A and B. Trio-binning has been used extensively to help phase and assemble animal genomes (Koren et al., 2018; Yen et al., 2020; Rhie et al., 2021; Yang et al., 2021; Rautiainen et al., 2023). In plants, it has recently been used for an inter-specific hybrid (Montgomery et al., 2020) and then most recently for a cross between subspecies (Huang et al., 2022), but not yet for an intra-specific cross. Intra-specific crosses, or breeding within the same species, represent most plants that researchers and breeders are working with.

In this study, we applied trio-binning to simultaneously assemble two phased genomes from an intra-specific cross of two parental chili pepper lines (*Capsicum annuum* L. cv ‘HDA149’ and cv ‘HDA330’). Accurate assembly of the chili pepper (*Capsicum annuum* L., 2n=2x=24) genome is frustrated by its large size (3.5 Gbp) and complexity due to high rate of repetitive elements (75-80%) (Kim et al., 2014; Qin et al., 2014; Hulse-Kemp et al., 2018; Lee et al., 2022; Shirasawa et al., 2022). Our results show that this method, when combined with modern scaffolding approaches, can successfully be used to produce two high quality phased genomes that are just as contiguous as the best currently available references, produced in recent pan-genome efforts (*Capsicum annuum* L. cultivars ‘Dempsey’ and ‘Zhangshugang’) (Lee et al., 2022; Liu et al., 2023). We provide best practices that can be applied by other groups seeking to produce quality biologically accurate, haplotype-resolved reference genomes for their lines of interest.

## 2 Results

### 2.1 Plant selection and sequencing

The two *Capsicum annuum* L. (2n = 2x = 24) parental pepper plants, HDA149 and HDA330, were confirmed as double haploids with the Illumina PepperSNP16K array (Hulse-Kemp et al., 2016). As expected, each line exhibited near complete homozygosity across all SNP sites in the array; 99.7% for HDA149 and 99.5% for HDA330 (**Supplemental Data 1**). Compared to each other, the parents had different alleles at 26.1% of SNP sites on the array and mummer alignments of the F_1_ indicated a genome-wide heterozygosity rate of 0.1168%.

We sequenced the parental plants at 45-50x depth with Illumina 150 bp paired end reads. The two parent plants were crossed to generate the F_1_ hybrid plant and it was sequenced to 58x depth with PacBio HiFi long reads (Sequel IIe) over 7 SMRT cells. Depth of sequencing and genome-wide heterozygosity were calculated based on the estimated genome size of *Capsicum annuum* of 3.5 Gb (Belletti et al., 1998; Moscone et al., 2003; Hulse-Kemp et al., 2018). The resulting three DNA sequence data sets (HDA149 Illumina short reads, HDA330 Illumina short reads, and the F_1_ Hifi reads) were used for trio-binning genome assembly.

### 2.2 Trio-binning assembly

We conducted trio-binning on the PacBio HiFi reads of the F_1_ hybrid using the two assembly softwares available at the time of this research, TrioCanu (Koren et al., 2018) and Hifiasm (Cheng et al., 2021). Both softwares utilize the k-mers from the parental short reads for haplotype partitioning. TrioCanu bins the HiFi reads prior to assembly; in contrast, Hifiasm partitions haplotigs after assembly. Of the 11,822,010 total HiFi reads after filtering, TrioCanu partitioned 4,586,239 reads (38.8%) to the HDA149 specific bin, 4,505,092 (38.1%) to the HDA330 specific bin, and 2,729,826 (23.1%) to the shared bin of non-haplotype specific reads. The shared reads and corresponding haplotype binned reads were used to generate a TrioCanu assembly for each parent. The resulting assemblies, TrioCanu HDA149 and TrioCanu HDA330, were highly contiguous with N50 values of 66.53 and 86.50 Mb, and genome size values of 3.31 and 3.30 Gb (**Table 1**). The Hifiasm assemblies, Hifiasm HDA149 and Hifiasm HDA330, exhibited higher contiguity with N50 values of 228.06 and 177.89 Mb, but lower genome size values of 3.10 and 3.09 Gb (**Table 1**).

**Table 1:** Experimental assembly comparison.

	Hifiasm HDA149	Hifiasm HDA330	TrioCanu HDA149	TrioCanu HDA330
**Binning software**	yak	yak	Canu v2.2	Canu v2.2
**Assembly software**	Hifiasm v0.16.1-r375	Hifiasm v0.16.1-r375	Canu v2.2	Canu v2.2
**Number of contigs**	364	119	5879	5914
**Contig N50 (Mb)**	228.056	177.885	66.526	86.496
**Longest contig (Mb)**	263.427	270.729	40.427	57.044
**Assembly Size (Mb)**	3100.1	3088.7	3306.1	3297.9

Comparison of genome assembly statistics of trio-binned assemblies generated with Hifiasm and HiCanu.

### 2.3 Haplotype switching

**Figure 2:**
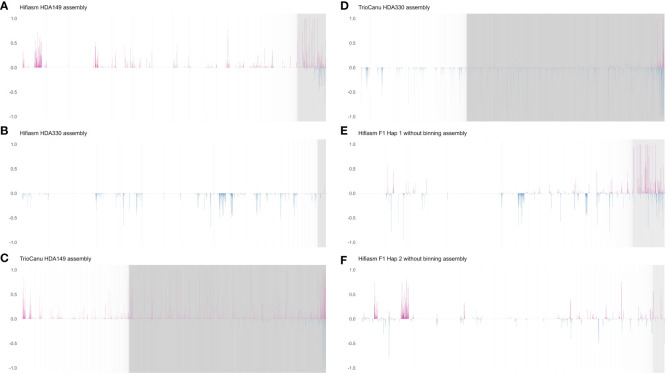
Haplotype switching. Haplotype switching was illustrated by aligning TrioCanu binned HiFi reads of parent A (HDA149) and parent B (HDA330) to each contig level genome assembly. The x-axis shows 1 Mb windows across contigs. The contigs were arranged from longest to shortest. Vertical gray lines show the boundaries of contigs. The y-axis shows the difference in percent coverage of the binned reads over a 1 Mb window of the given assembly. Higher coverage of HDA149 is shown in pink and higher coverage of HDA330 is shown in blue. **(A)** Hifiasm HDA149 assembly with trio-binning. **(B)** Hifiasm HDA330 assembly with trio-binning. **(C)** TrioCanu HDA149 assembly with trio-binning. **(D)** TrioCanu HDA330 assembly with trio-binning. **(E)** Hifiasm haplotype 1 assembly in default run mode, without parental k-mers for trio-binning. **(F)** Hifiasm haplotype 2 assembly in default run mode, without parental k-mers for trio-binning.

To confirm that the trio-binning assemblies were haplotype resolved, we mapped the TrioCanu binned reads onto each of the assemblies and calculated differences in alignment coverage over 1 Mb windows. Haplotype specific windows of an assembly will show high alignment coverage for the corresponding set of parent specific binned reads and low coverage for the opposite set of parent specific binned reads. Each assembly should show differences in alignment rates favoring only the reads from their specific corresponding bins if there is no haplotype switching, for example TrioCanu HDA149 should have windows of higher alignment rates only for HDA149 reads. All four assemblies, TrioCanu HDA149 (97.7%), TrioCanu HDA330 (97.7%), Hifiasm HDA149 (97.7%) and Hifiasm HDA330 (99.3%) showed windows of higher alignment rates for their corresponding haplotype bin, indicating that the assemblies were correctly haplotype resolved (**Figures 2A–D**).

As a control, we also generated non-binned Hifiasm assemblies of the F_1_ and calculated differences in alignment coverage of the binned reads. By default, without parental k-mers or Hi-C data, Hifiasm attempts to naively phase haplotypes and produces 3 assemblies: primary, hap1 and hap2 https://github.com/chhylp123/hifiasm, Accessed 03/09/2023). These non-binned assemblies showed haplotype switching, calculated in the same way as above, the Hifiasm hap1 had 86.8% and hap2 had 87.5% alignment rates. This was visualized as 1 Mb windows which showed alternating haplotypes of higher alignment rate (**Figures 2E, F**).

### 2.4 Assembler comparison

In total, we generated 4 trio-binned assemblies with two assemblers (Hifiasm and HiCanu). We named these assemblies ‘TrioCanu HDA149’, ‘TrioCanu HDA330’, ‘Hifiasm HDA149’ and ‘Hifiasm HDA330’ (**Table 1**). The Hifiasm assemblies had 16-50x fewer contigs and 2-3x higher N50 values than the TrioCanu assemblies (**Table 1**), but the TrioCanu assemblies were ~ 200 Mb larger in size.

**Figure 3:**
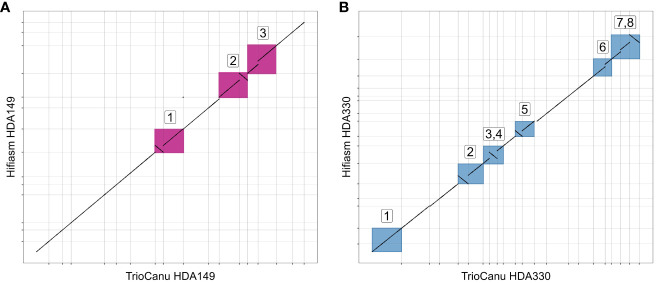
Utility of reciprocal scaffolding of assemblies from alternate software. Dotplots show alignments between largest contigs of TrioCanu and Hifiasm assemblies. Opportunities to improve contiguity through iterative scaffolding are highlighted in boxes that are numbered and shown in pink for HDA149 **(A)** or blue for HDA330 **(B)**.

We were curious if the two assemblers differed in their ability to assemble the same genomic regions. To test this, we mapped the TrioCanu assemblies against the Hifiasm assemblies and generated dotplots of the largest contigs, > 5 Mb. Overall, the two assemblers generated the same large contigs, however there were a number of sequence regions where one was able to assemble through while the other was not. In HDA149, Hifiasm was able to assemble through 3 regions that TrioCanu was not (**Figure 3A**). In HDA330, Hifiasm assembled though 7 regions that TrioCanu did not, and TrioCanu assembled through 1 region that Hifiasm did not (**Figure 3B**). Given that in these 11 regions one assembler performed better than the other, we decided to leverage this information during our scaffolding workflow, described in the next section.

### 2.5 Scaffolding and quality assessment of assemblies

The assemblies were highly contiguous owing to the PacBio HiFi reads, but we were interested to see if our data could generate chromosome scale *de novo* assemblies. To achieve this, we built an iterative scaffolding workflow that first used homology scaffolding between the different assemblers followed by gap-filling with RagTag software (**Figure 1B** and **Supplemental Table 1**). For example, Hifiasm HDA330 was scaffolded against and gap-filled using TrioCanu HDA330. Step 1 increased scaffold N50 values from 177.8 to 247.4 Mb for Hifiasm HDA330, from 86.5 to 232.1 Mb for TrioCanu HDA330, and from 66.5 to 231.1 Mb for TrioCanu HDA149 (**Supplemental Data 2**). As expected, Hifiasm HDA149 assembly N50 values did not increase in this step as there were no regions that TrioCanu had assembled better than Hifiasm in this haplotype (**Figure 3A**). However, during gap-filling, contig N50 values did increase for all 4 assemblies. For the third step, we anchored the assemblies using the other haplotype. For example, Hifiasm HDA330 was used to scaffold Hifiasm HDA149. Improvements were made in scaffold N90 values, from 134.5 Mb to 243.7 Mb in TrioCanu HDA149, from 131.9 to 189.9 Mb in Hifiasm HDA149, from 178.1 to 237.8 Mb in TrioCanu HDA330, and 177.9 to 189.9 Mb in Hifiasm HDA330. Although N50 and N90 values had improved considerably, the majority of each assembly (>3.0 Gb) was still not yet captured in 12 scaffolds representing each of 12 chromosomes (**Supplemental Data 2**, page “Scaffolding Statistics”).

Next, we merged the Bionano optical map of the F_1_ sample with each of the four contig level assemblies. Optical mapping gave scaffold N50 values between 182.6 – 210.9 Mb, however, during conflict resolution, the Bionano Saphyr software also made between 26 – 47 cuts to the contigs of our assemblies (**Supplemental Data 2**, page “Scaffolding Statistics”). This substantially lowered the contig N50 values by 2-3x. To retain contig integrity, we anchored the Step 3 scaffolded assemblies onto their respective Bionano-Hybrid assembly using RagTag (**Figure 3**). This brought our assemblies closer to full chromosome scale, with ~ 3.0 Gb of the assemblies being captured in 13 scaffolds for TrioCanu HDA149 and Hifiasm HDA149 and in 14 scaffolds for TrioCanu HDA330 and Hifiasm HDA330.

Final assembly of pseudomolecules were oriented and given chromosome designations through RagTag homology scaffolding to the previously published pepper assembly, Dempsey v1.0 (**Figure 1B** and **Supplemental Table 1**). During this step, we found that using Dempsey v1.0 allowed us to anchor distal ends of chr5 and chr11 in our assemblies. Our final Hifiasm assemblies which had the best assembly statistics (**Table 2**) were chosen as the final reference assemblies, HDA149v1.0 and HDA330v1.0, are available through NCBI JAVHYQ000000000 and JAVHYR000000000, respectively and at the SolGenomics database (https://solgenomics.net/ftp/genomes/Capsicum_annuum/C.annuum_F1_HDA149_x_HDA330).

**Table 2:** Final assembly statistics.

	HDA149v1.0	HDA330v1.0	Zhangshugang	Dempsey	UCD10X
**Contig number**	359	112	91	532	134,101
**Scaffold number**	239	51	601	121	81,378
**Contig N50 (Mb)**	228.1	228.6	35.4	18.3	0.1
**Contig L50**	7	7	25	51	6,631
**Scaffold N50 (Mb)**	266.0	281.3	259.7	260.5	227.2
**Contig N90 (Mb)**	131.9	171.4	19.4	9.7	0.1
**Contig L90**	10	10	49	98	13,035
**Scaffold N90 (Mb)**	254.0	254.6	253.2	249.5	219.1
**Assembly Size (Mb)**	3,100.6	3,118.8	3,023.8	3,053.5	3,124.3
**% of Estimated Genome Size**	88.6%	88.25%	86.39%	86.7%	89.3%
**% of Assembly Placed in Chromosomes**	99.6%	99.8%	99.9%	99.7%	83.2%
**Busco Completeness (%)**	97.4%	98.4%	97.1%	97.7%	96.5%
**LAI**	8.98	9.00	8.19	7.70	6.79
**Source**	This study	This study	Liu et al., 2023	Lee et al., 2022	Hulse-Kemp et al., 2018
**Sequencing technology**	PacBio HiFi	PacBio HiFi	PacBio CLR and Illumina short reads	PacBio CLR and Illumina short reads	10x Genomics Linked-Reads
**Scaffolding technology**	Bionano Optical Maps and RagTag homology based scaffolding	Bionano Optical Maps and RagTag homology based scaffolding	Phase Genomics Hi-C	Dovetail Hi-C, Bionano Optical Maps and four genetic maps	Four genetic maps, three transcriptome maps, and one genomic map

Comparison of assembly statistics between our trio-binned final assemblies, HDA149v1.0 and HDA330v1.0 and three previously published assemblies, Zhangshugang v1.0, Dempsey v1.0 and UCD10x v1.0.

Our final TrioCanu assemblies, HDA149alt-v1.0 and HDA330alt-v1.0, are available through USDA Ag Data Commons (**Supplemental Table 2**, https://data.nal.usda.gov/dataset/triobinning-capsicum-annuum-genome-assemblies).

**Figure 4:**
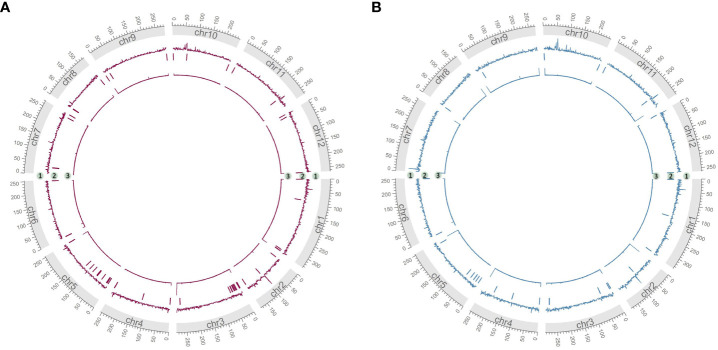
Characterization of developed assemblies. Circos plots of final Hifiasm assemblies HDA149v1.0 **(A)** and HDA330v1.0 **(B)** show long terminal repeat content across 1 MB windows in track 1, gap locations in track 2 and telomere repeat peaks across 1 kb windows in track 3.

As measurements of assembly quality, we examined gaps in the Hifiasm assemblies, repeat content and telomere repeats (**Figures 4A, B**). We saw that generally gaps occurred toward the telomeric regions of the chromosomes, coinciding with the fact that most chromosomes were completely captured in a single contig as seen in chr7 of HDA330 or nearly completely captured as seen in chr6, chr8, chr9, chr10 and chr12 of HDA330. Strong telomere repeat peaks were detected in 12 of the 24 chromosome arms of HDA330. Peaks in long-terminal repeat (LTR) content did not mandate gaps in the assemblies, as seen clearly in chr1, chr7 and chr10 of HDA330. Similar results were seen for HDA149.

**Figure 5:**
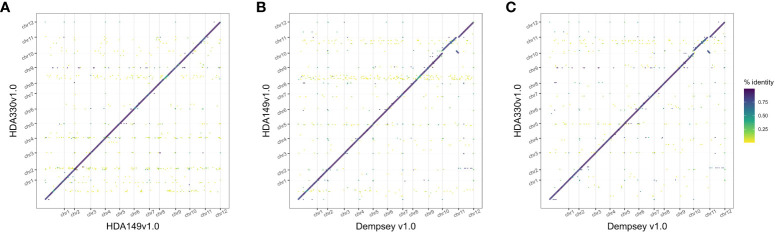
Comparison of final Hifiasm assemblies. Dotplots of assembly by assembly alignments of **(A)** HDA149v1.0 to HDA330v1.0, **(B)** HDA149v1.0 to Dempsey v1.0, and **(C)** HDA330v1.0 to Dempsey v1.0. Gridlines show boundaries of chromosomes (x-axis) and color indicates percent identity of the alignment.

The final Hifiasm assemblies, HDA149v1.0 and HDA330v1.0, had minimal large scale structural variation (**Figure 5A**). Divergences in percent sequence identity were observed on several chromosomes, in particular chr9, chr7, and chr1. These results were expected given that HDA149 and HDA330 were developed as resistance gene introgressions into the Yolo Wonder background (Hendy et al., 1985). Compared to Dempsey, there was a large inversion on chr11 in both assemblies (**Figures 5B, C**). Overall, trio-binning with Hifiasm produced haplotype level assemblies with substantially higher contig N50 values of 228 Mb compared to 18 Mb for Dempsey and 35.4 Mb for Zhangshugang (**Table 2**). The trio-binning assemblies also had higher long-terminal repeat assembly index (LAI) scores of 8.98 and 9.00 compared to 7.70 for Dempsey and 8.19 for Zhangshugang (**Table 2**) (Lee et al., 2022; Liu et al., 2023). Our assemblies captured 77.0 and 92.5 Mb more of the total *C. annuum* genome. Additionally, the HDA330 assemblies reported a slight increase in genic space coverage as estimated with BUSCO (**Table 2**).

## 3 Discussion

We generated two high quality, fully haplotype phased *de novo* pepper (*Capsicum annuum* L.) genome assemblies using trio-binning, as evidenced by LAI scores of 8.98 and 9.00. These assemblies accurately represent the haploid genomes within a single diploid intra-specific hybrid plant, making a 9.6-9.8% improvement on completeness based on genome size estimates and are 6.4X more contiguous at the contig level with over 90% of the assembly sequence (Contig L90, **Table 2**) included in the first 10 contigs of each of the haplotypes produced in this study. High quality pepper genome assemblies such as the two presented here and those already available create a valuable community resource for in-depth analysis of genome evolution, structural variation, and haplotype specific gene clusters. Of particular interest in crop breeding are resistance gene clusters that are often haplotype specific with little or no recombination due to significant structural variation (Jiao and Schneeberger, 2020; Vaughn et al., 2022). A single reference genome inhibits characterization of these resistance genes and hinders reliable molecular marker development. In this new era of project specific high quality genome assemblies, researchers can now easily capture these important haplotype specific regions.

Generating *de novo* assemblies of an F_1_ individual is a powerful tool for biparental mapping, experimental population studies, and breeding. These assemblies capture the complete landscape of sequence diversity segregating in that population, which is often difficult to discern when using a generic reference genome. An excellent example highlighting the improved ability of having the complete landscape of sequence diversity for detecting causative loci for traits of interest was recently published in melon (Vaughn et al., 2022). However, separately assembling two parental long read assemblies is more costly and potentially more error prone than trio-binning. Another benefit of trio-binning is that its ability to partition haplotypes increases with increasing heterozygosity of the individual, as shown in outbred individuals such as humans (Koren et al., 2018) and *Arctia plantaginis* (Yen et al., 2020), and in subspecies F_1_ hybrids such as *Bos taurus taurus* x *Bos taurus indicus* (Koren et al., 2018) and *Amaranthus tuberculatus* x *Amaranthus hybridus* (Montgomery et al., 2020). The utility of Bionano optical maps have been extensively demonstrated by the Telomere-to-Telomere (T2T) Consortium for human genomes (Mc Cartney et al., 2022) and here we showed that Bionano optical maps can also be used in conjunction with trio-binning in plants.

We found benefit from utilizing multiple assembly softwares and the best solution was to use components from both software. The algorithms perform differently in different parts of the genomes and can complement each other through scaffolding techniques (**Figure 3**). Future development of these two softwares may improve usability and results that may negate the strategy we found performed the best. But if time and compute resources allow, it may also be beneficial for others to generate assemblies from both softwares so that iterative scaffolding can exploit the differences in assembler software and improve genome contiguity. Integration of other techniques such as HiC may also help to improve assembly of missing components into final scaffolds and has been shown to enable some haplotype-based assembly (Cheng et al., 2022), but would require additional cost and is difficult to obtain high-quality data in many plants.

**Figure 6:**
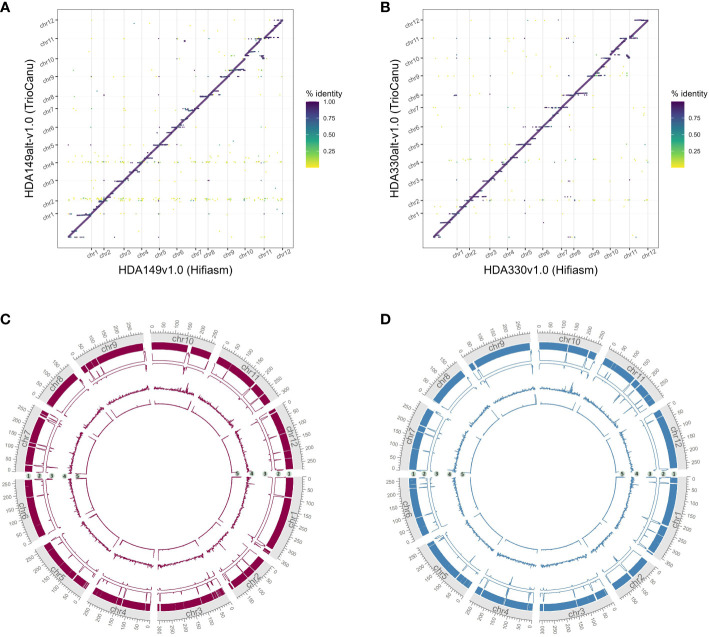
Comparison of final TrioCanu and Hifiasm assemblies. Dotplots of assembly by assembly alignments of **(A)** HDA149alt-v1.0 (TrioCanu) to HDA149v1.0 (Hifiasm), **(B)** HDA330alt-v1.0 (TrioCanu) to HDA330v1.0 (Hifiasm). Gridlines show boundaries of chromosomes (x-axis) and color indicates percent identity of the alignment. Circos plot of final TrioCanu assemblies HDA149alt-v1.0 **(C)** and HDA330alt-v1.0 **(D)** show regions of shared sequence to the corresponding final Hifiasm assembly in track 1, the number of gaps across 1 MB windows in track 2, the HiFi read alignment coverage across 1 MB windows in track 3, long terminal repeat content across 1 MB windows in track 4, and telomere repeat peaks across 1 kb windows in track 5.

Careful consideration, and likely direct comparison, of available assembly software should be made when generating *de novo* assemblies. Approximately 200 Mb additional of sequence was captured into the final chromosomes of the TrioCanu assemblies compared to the Hifiasm assemblies (**Figure 6**). These additional sequences appeared repetitive given that they aligned across the Hifiasm assembly (**Figures 6A, B**). The additional regions were highly fragmented as shown by the number of gaps in the assemblies (**Figures 6C, D**, track 2) and likely contain assembly errors given the decrease in HiFi read coverage (**Figures 6C, D**, track 3). TrioCanu better assembled telomeric regions (**Figures 6C, D**, track 5), these results suggest that Hifiasm may be collapsing repetitive regions compared to TrioCanu. Resolution of complex repetitive regions have been achieved through a combination of several technologies and softwares for the human genome (Nurk et al., 2022). Cost and time of achieving a telomere-to-telomere genome assembly must be weighed against the research needs of each project.

This work shows that *de novo* assemblies using trio-binning as developed in this study are now relatively inexpensive and easy to generate even for intra-specific hybrids (in this case, ~$15,000 cost at the time of data generation for the raw reagent cost of sequencing and Bionano) and becoming even more feasible with continual drops in sequencing costs. Plant researchers should consider using trio-binning with the methods outlined here in future studies to represent the true biology of their plants to obtain haplotype-resolved genomes.

## 4 Materials and methods

### 4.1 Hybrid development and identification

Two *Capsicum annuum* L. double haploid lines were selected as parents for generating a controlled cross, HDA149 and HDA330 (Hendy et al., 1985; Thies and Ariss, 2009). A single individual of each parental line was used to make a cross HDA149 x HDA330. Young leaf tissue (two to three unfurled leaves) from both parental lines was extracted using a DNeasy Plant kit (Qiagen, Hilden, Germany). DNA was quantitated using a Nanodrop spectrophotometer (Thermofisher Scientific, Waltham, MA, USA). Each plant was genotyped using the PepperSNP16K array (Hulse-Kemp et al., 2016). As parents were confirmed to be double haploids using the array, uniform F_1_ individuals were utilized downstream in combination with the two single parental plants to represent a trio (mother-father-offspring).

### 4.2 Parental sequencing

The double haploid parents, HDA149 and HDA330 were sequenced with short read sequencing. TruSeq PCR-free libraries were prepared and the samples were run on the Illumina Novaseq 6000 (Illumina, San Diego, CA, USA) which generated 150 bp paired end reads. The raw sequencing coverage of HDA149 was 49.3x and coverage of HDA330 was 45.3x. Raw sequencing data is available through the NCBI sequence read archive under SRR21710630 (HDA149) and SRR21710629 (HDA330).

The quality of the Illumina sequencing data was checked with FastQC version 0.11.9 (Andrews, 2010). Fastp v0.21.0 (Chen et al., 2018) was used to trim the first 12 bp, as well as remove any poly-g tails and adapter sequences. A minimum length of 50 bp was also required for a read to pass quality filtering. The resulting coverage for HDA149 was 45.0x and 41.4x for HDA330.

### 4.3 Hybrid sequencing

Uniform F_1_ hybrid individuals (HDA149 x HDA330) were grown in greenhouse conditions then dark treated for 48 hours, unexpanded leaf tissue was flash frozen in liquid nitrogen. Nuclei were isolated from 1 gram of young leaf tissue using the Bionano Prep Plant Tissue DNA Isolation kit (Bionano Genomics, San Diego, CA). Subsequently, high molecular weight (HMW) genomic DNA was extracted from the nuclei using the Circulomics Nanobind Plant Nuclei Big DNA Kit (Pacific Biosciences, Menlo Park, CA). HMW DNA was sheared with the Covaris g-TUBE (Woburn, MA) to target fragments near 15Kb. Sheared HMW DNA was used to prepare a PacBio SMRTbell library and size selected using the BluePippin (Sage Science, Beverly, MA). The library was sequenced on 7 cells of the Sequel IIe (Pacific Biosciences, Menlo Park, CA), generating 11,829,089 PacBio HiFi reads equivalent to 58x coverage of the 3.5 Gb *C. annuum* L. genome. The PacBio HiFi reads are available through the NCBI sequence read archive under BioProject PRJNA884326. HiFiAdapterFilt identified and removed ~0.05% of PacBio HiFi reads that had adapter contamination (https://github.com/sheinasim/HiFiAdapterFilt, accessed 2023).

### 4.4 Bionano optical mapping

Optical mapping was done with ultra HMW genomic DNA of the F_1_. Briefly, specific genomic sequences were fluorescently labeled with the Direct Label Enzyme-1 of the Bionano Prep Direct Label and Stain (DLS) kit (Bionano Genomics, San Diego, CA, USA) and imaged using the Saphyr system (Bionano Genomics, San Diego, CA, USA).

### 4.5 Genome assembly

Genomes were assembled using the two trio-binning softwares available as of 2022, TrioCanu (Koren et al., 2018) and Hifiasm (Cheng et al., 2021). TrioCanu and Hifiasm differ in their trio-binning approach, with TrioCanu binning the HiFi reads prior to assembly and Hifiasm binning contigs after assembly. Both rely on distinct parental k-mers from short reads to bin the long reads or contigs. Briefly, TrioCanu takes the trimmed parental short reads as input under the ‘-haplotype’ option and finds haplotype-distinct 21-mers with the k-mer counting software meryl (Rhie et al., 2020). The alignment of the haplotype-distinct 21-mers to HiFi reads is used to determine to which bin a given HiFi read belongs, i.e. to parental haplotype HDA149 or HDA330. If a haplotype cannot be confidently assigned to either haplotype then the read is placed in an ‘unknown’ fasta bin. Given that HiFi reads have low sequencing errors, these unknown reads are primarily the shared sequences between the two haplotypes. TrioCanu results in three fasta files, parental haplotype 1, parental haplotype 2 and unknown haplotype (shared). Alternatively, Hifiasm applies a similar principle to assembled contigs (haplotigs) to partition into the corresponding parental haplotype.

TrioCanu v2.2 does not yet directly support genome assembly with HiFi reads, but HiCanu (Nurk et al., 2020) does. Therefore, binning and assembly were run in two steps, the first with TrioCanu and the second with HiCanu. HiFi read binning was accomplished with ‘canu -p binned_reads -d binned_reads -haplotypeHDA149 illumina-HDA149*fq.gz -haplotypeHDA330 illumina-HDA330*fq.gz -pacbio HiFi-reads*fq.gz’. TrioCanu stops after binning because the HiFi reads appear to be corrected CLR reads. In the second step, assemblies are made with ‘canu -p TrioCanu_HDA149_assembly -d TrioCanu_HDA149_assembly genomeSize=3.5g -pacbio-hifi binned_reads/haplotype/haplotype-HDA149.fasta.gz binned_reads/haplotype/haplotype-unknown.fasta.gz’. The same script was run for HDA330, but with the corresponding HDA330 binned reads. For simplicity, we refer to these assemblies as TrioCanu-HDA149 and TrioCanu-HDA330.

Parental k-mers for Hifiasm are first generated with yak v0.1(r56) (https://github.com/lh3/yak, accessed 2023) using ‘count -k31 -b37’ settings. Yak does not support multiple input files so it is necessary to first concatenate all sequence files for a parent into a single file. The yak dumps are supplied to Hifiasm version 0.16.1-r375 for assembly of the HiFi reads into the two haplotypes with the command ‘hifiasm -1 HDA149.yak -2 HDA330.yak HiFi-reads*fq.gz’. We refer to the resulting assemblies Hifiasm HDA149 and Hifiasm HDA330.

For comparison, we also generated a Hifiasm assembly without trio-binning because the software attempts to partition haplotigs even without parental k-mers to inform it. The script was ‘hifiasm HiFi-reads*fq.gz’. We called these assemblies Hifiasm-F1-Hap1 and Hifiasm-F1-Hap2.

### 4.6 Scaffolding

The four assemblies were scaffolded with the F_1_ Bionano optical map and designated as Hifiasm-HDA149.BN, Hifiasm-HDA330.BN, TrioCanu-HDA149.BN and TrioCanu-HDA330.BN. Scaffolding of contigs was accomplished in 5 steps using an iterative scaffolding workflow (**Figure 1B**, **Supplemental Table 1**). Step 1 leveraged differences in the two assembly softwares by reciprocally scaffolding TrioCanu assemblies to Hifiasm assemblies using Ragtag v2.1.0 ‘scaffold’ option and default parameters (Alonge et al., 2022). Step 2 patched gaps in assemblies using Ragtag ‘patch’ with minimap2 as the aligner. Step 3 scaffolded the alternative parent haplotype assemblies against each other using Ragtag ‘scaffold’. Step 4 scaffolded the assemblies to the corresponding Bionano contig-assembly hybrid scaffolds using Ragtag ‘scaffold’. Step 5 scaffolded assemblies to Dempsey v1.0 (Lee et al., 2022) to order and orient chromosomes using RagTag ‘scaffold’. The resulting assemblies became the publicly released versions.

### 4.7 Analysis and visualization

Genome statistics were retrieved using stats.sh of the BBTools suite version 38.79 (Bushnell, 2022). BUSCO v5.2.2 in genome mode with the embryophyta_odb10 database was used for calculating completeness scores (Manni et al., 2021). Long-terminal repeat assembly index (LAI) values were found using the LAI software with default parameters, analysis was run locally as well as using the webportal at https://bioinformatics.um6p.ma/PlantLAI/lai-pipeline (accessed 8/2023) (Ou et al., 2018; Mokhtar and Allali, 2022; Mokhtar et al., 2023).

Trio-binning and scaffolding workflow figures (**Figure 1**) were made in BioRender.com.

Haplotype switching (**Figure 2**) in assemblies was determined by aligning TrioCanu binned reads (HDA149 and HDA330) to the given assembly with minimap2 version 2.24-r1122 (Li, 2018). Assembly files were indexed with samtools version 1.9 (Danecek et al., 2021) and 1 Mb windows were made with bedtools version 2.30.0 ‘makewindows’(Quinlan and Hall, 2010). Read coverage for each window was calculated with bedtools ‘coverage’. The difference in percent coverage of each window for HDA149 and HDA330 binned reads was calculated in RStudio version 2022.07.2 + 576 (RStudio Team, 2020) with R version 4.2.1 (R Core Team, 2022) and plotted with ggplot2 version 3.3.6 (Wickham, 2016). Large positive differences in coverage meant HDA149 reads covered more of the window than HDA330 reads did and therefore that the region is haplotype specific to HDA149.

Sample specific telomere repeats were identified with the ‘explore’ function of Telomere Identification Toolkit, tidk (https://github.com/tolkit/telomeric-identifier, accessed 2023) on the final genome assemblies with a minimum string length of 5 and maximum length of 12. The top hits ‘AAAAATAGTAG’ and ‘TTAGGG’ were searched in the final genome assemblies with default settings of the tidk ‘search’ function. LTRharvest v2.9.4 of Genome Tools (Gremme et al., 2013), with specifications of ‘-minlenltr 100 -maxlenltr 7000 -mintsd 4 -maxtsd 6 -motif TGCA -motifmis 1 -similar 85 -vic 10 -seed 20 -seqids yes’ was used to annotate long terminal repeat retrotransposons in the final assemblies. Seqkit v0.10.1 (Shen et al., 2016) ‘locate’ was used to find gaps in assemblies by searching for strings of Ns. LTR content over 1Mb windows, telomere repeat counts over 1 kb windows, and gap locations were visualized with the circlize package (Gu et al., 2014) in R.

Dotplots in **Figures 3**, **5**, **6** were generated using minimap2 and modified dotplotly code (https://github.com/tpoorten/dotPlotly, accessed 2022) in RStudio with R. The dotplotly code uses the R packages dplyr version 1.0.10 (Wickham et al., 2023) and ggplot2 (Wickham, 2016). Scripts for figures can be found at the Github repository https://github.com/USDA-ARS-GBRU/Pepper_TrioBinning/.

Genome wide heterozygosity was calculated as the number of single nucleotide polymorphism and insertion/deletion positions from unique alignments between the final Hifiasm assemblies HDA149v1.0 and HDA330v1.0. Alignments were made with the default settings of nucmer in mummer v4.0.0rc1 (Marçais et al., 2018). Variant positions of unique alignments were called from the delta file with ‘show-snps -C’ of mummer v4.0.0rc1.

## Data availability statement

The datasets presented in this study can be found in online repositories. The names of the repository/repositories and accession number(s) can be found in the article/**Supplementary Material**.

## Author contributions

EED, WBR, AMH-K conceived the project. RCY, SAS, BES performed sequencing and generated raw data. EED analyzed data and wrote the manuscript. ANS and RCY participated in data analysis. WBR and AMH-K supervised the project. All authors contributed to the article and approved the submitted version.

## Supplementary material

Supplementary Data Sheet 1 Genotyping data from parental samples on the PepperSNP16K array.

Supplementary Data Sheet 2 Detailed assembly statistics.

Supplementary Table 1 Scaffolding workflow details.

Supplementary Table 2 Final assembly availability links.
