# Supplementary material for: Representing true plant genomes: haplotype-resolved hybrid pepper genome with trio-binning
Source: Front Plant Sci. 2023 Nov 16;14:1184112. doi: 10.3389/fpls.2023.1184112 (PMC10687446; doi:10.3389/fpls.2023.1184112)
Supplement: Supplementary Table 1 — Scaffolding workflow details. [file Table_1.docx]

**Supplemental Table 1**

Detailed outline of the 5 scaffolding steps taken to produce final assemblies. Steps are visually shown on Figure 1 of the trio-binning workflow. After final quality control, the outputs of Step 5 were renamed as outlined in Supplemental Table 2 for release of v1.0 assemblies.

| **Step** | **Description** | \| **Query** \| **Reference** \| **Output** \| \| --- \| --- \| --- \| |
| --- | --- | --- | --- | --- | --- |
| **Step 1** | Homology based scaffolding between Hifiasm and TrioCanu assemblies | \| Hifiasm-HDA149 \| + \| TrioCanu-HDA149 \| = \| Hifiasm-HDA149.1 \| \| --- \| --- \| --- \| --- \| --- \| \| TrioCanu-HDA149 \| + \| Hifiasm-HDA149 \| = \| TrioCanu-HDA149.1 \| \| Hifiasm-HDA330 \| + \| TrioCanu-HDA330 \| = \| Hifiasm-HDA149.1 \| \| TrioCanu-HDA330 \| + \| Hifiasm-HDA330 \| = \| TrioCanu-HDA330.1 \| |
| **Step 2** | Homology based gap filling between Hifiasm and TrioCanu assemblies | \| Hifiasm-HDA149.1 \| + \| TrioCanu-HDA149.1 \| = \| Hifiasm-HDA149.2 \| \| --- \| --- \| --- \| --- \| --- \| \| TrioCanu-HDA149.1 \| + \| Hifiasm-HDA149.1 \| = \| TrioCanu-HDA149.2 \| \| Hifiasm-HDA330.1 \| + \| TrioCanu-HDA330.1 \| = \| Hifiasm-HDA149.2 \| \| TrioCanu-HDA330.1 \| + \| Hifiasm-HDA330.1 \| = \| TrioCanu-HDA330.2 \| |
| **Step 3** | Homology based scaffolding to between HDA149 and HDA330 | \| Hifiasm-HDA149.2 \| + \| Hifiasm-HDA330.2 \| = \| Hifiasm-HDA149.3 \| \| --- \| --- \| --- \| --- \| --- \| \| TrioCanu-HDA149.2 \| + \| TrioCanu-HDA330.2 \| = \| TrioCanu-HDA149.3 \| \| Hifiasm-HDA330.2 \| + \| Hifiasm-HDA149.2 \| = \| Hifiasm-HDA149.3 \| \| TrioCanu-HDA330.2 \| + \| TrioCanu-HDA149.2 \| = \| TrioCanu-HDA330.3 \| |
| **Step 4** | Homology based scaffolding to Bionano-contig hybrid scaffolds | \| Hifiasm-HDA149.3 \| + \| Hifiasm-HDA330.BN \| = \| Hifiasm-HDA149.4 \| \| --- \| --- \| --- \| --- \| --- \| \| TrioCanu-HDA149.3 \| + \| TrioCanu-HDA330.BN \| = \| TrioCanu-HDA149.4 \| \| Hifiasm-HDA330.3 \| + \| Hifiasm-HDA149.BN \| = \| Hifiasm-HDA149.4 \| \| TrioCanu-HDA330.3 \| + \| TrioCanu-HDA149.BN \| = \| TrioCanu-HDA330.4 \| |
| **Step 5** | Homology based scaffolding to Dempsey v1.0 | \| Hifiasm-HDA149.4 \| + \| Dempsey v1.0 \| = \| Hifiasm-HDA149.5 \| \| --- \| --- \| --- \| --- \| --- \| \| TrioCanu-HDA149.4 \| + \| Dempsey v1.0 \| = \| TrioCanu-HDA149.5 \| \| Hifiasm-HDA330.4 \| + \| Dempsey v1.0 \| = \| Hifiasm-HDA149.5 \| \| TrioCanu-HDA330.4 \| + \| Dempsey v1.0 \| = \| TrioCanu-HDA330.5 \| |
