# Supplementary material for: Representing true plant genomes: haplotype-resolved hybrid pepper genome with trio-binning
Source: Front Plant Sci. 2023 Nov 16;14:1184112. doi: 10.3389/fpls.2023.1184112 (PMC10687446; doi:10.3389/fpls.2023.1184112)
Supplement: Supplementary Table 2 — Final assembly availability links. [file Table_2.docx]

**Supplemental Table 2: Data availability**

| **Experimental Assembly name** | **Published assembly name** | **Availability** |
| --- | --- | --- |
| Hifiasm-HDA149.5 | Capsicum_annuum_HDA149v1.0 | NCBI: PRJNA944648  Genome: JAVHYQ000000000  SolGenomics: https://solgenomics.net/ftp/genomes/Capsicum_annuum/C.annuum_F1_HDA149_x_HDA330 |
| Hifiasm-HDA330.5 | Capsicum_annuum_HDA330v1.0 | NCBI: PRJNA987740  Genome: JAVHYR000000000  SolGenomics: https://solgenomics.net/ftp/genomes/Capsicum_annuum/C.annuum_F1_HDA149_x_HDA330 |
| TrioCanu-HDA149.5 | Capsicum_annuum_HDA149alt-v1.0 | USDA Ag Data Commons: https://data.nal.usda.gov/dataset/triobinning-capsicum-annuum-genome-assemblies |
| TrioCanu-HDA330.5 | Capsicum_annuum_HDA330alt-v1.0 | USDA Ag Data Commons: https://data.nal.usda.gov/dataset/triobinning-capsicum-annuum-genome-assemblies |
| **raw data** | | **Availability** |
| PacBio HiFi sequences for F1 (HDA149 x HDA330) | | NCBI BioProject PRJNA884326 |
| Illumina 150 bp paired-end sequencing data for HDA149 | | NCBI SRA SRR21710630 |
| Illumina 150 bp paired-end sequencing data for HDA330 | | NCBI SRA SRR21710629 |
